# Ultrastructural aspects of the skin in lipoid proteinosis (Urbach-Wiethe disease)^☆^^☆☆^

**DOI:** 10.1016/j.abd.2021.04.010

**Published:** 2021-09-17

**Authors:** Hiram Larangeira de Almeida, Raphael Goveia Rodeghiero, Patrícia Naomi Ando Suzuki, Marília Marufuji Ogawa

**Affiliations:** a Post-Graduation in Health and Behavior, Universidade Católica de Pelotas, Pelotas, RS, Brazil; b Department of Dermatology, Universidade Federal de Pelotas, Pelotas, RS, Brazil; c Department of Dermatology, Universidade Federal da São Paulo, São Paulo, SP, Brazil

**Keywords:** Lipoid proteinosis of Urbach and Wiethe, Microscopy, electron, scanning, Microscopy, electron, transmission, Skin diseases, genetic

## Abstract

Lipoid proteinosis is a rare autosomal recessive disease, characterized by hyaline deposits of PAS-positive material in tissues due to mutations in the *ECM1* gene. This study evaluated the ultrastructure of the skin of a 6-year-old child affected by this condition. The light microscopy identified PAS-positive hyaline deposits, which were more intense in the papillary dermis. Scanning electron microscopy of the dermis showed a compact papillary dermis and fibrillar deposits in the middle dermis. Transmission electron microscopy clearly showed the deposition of fibrillar material in the dermis, forming clusters adherent to elastic fibers, between the collagen bundles and the collagen fibers, and also filling up the cytoplasm of dermal fibroblasts.

## Introduction

Lipoid proteinosis (OMIM 247100), also called Urbach-Wiethe disease, or *hyalinosis cutis et mucosae*, is a rare genodermatosis, with an autosomal recessive pattern of inheritance, with varied expression, which may compromise multiple systems, such as the mucosa and internal organs, showing important cutaneous involvement.^1^

Its occurrence is due to mutations in the extracellular matrix protein 1 (ECM1) gene.^2^, ^3^ Cutaneous involvement is characterized by deposition of hyaline material in the papillary dermis, best seen with periodic acid Schiff (PAS) staining.

In its classic clinical picture, which has a chronic and benign course, patients exhibit signs and symptoms since childhood, including hoarseness and phonation difficulty (due to laryngeal involvement), followed by skin fragility and varioliform lesions. There is also neurological involvement due to the presence of calcifications in the central nervous system, with amigdala involvement being considered a pathognomonic sign.

This disease, despite being benign in most individuals, does not have a definitive treatment, which consists of symptomatic approaches in affected patients.

This is the case of a 6-year-old Caucasian boy, followed since the prenatal period for myelomeningocele, who was referred to the Department of Dermatology due to findings consistent with lipoid proteinosis on a control skull tomography.

**Figure 1:**
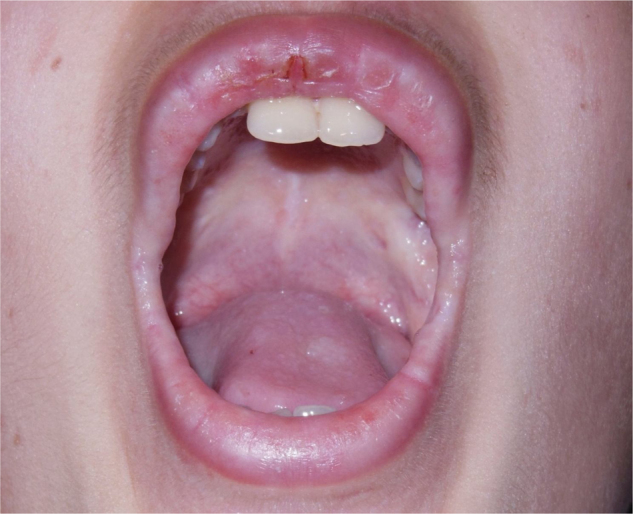
Clinical aspect – xanthomatous plaques on the palate.

The patient had shown low crying since birth and hoarseness when learning to speak, with a gradual worsening in recent years. The dermatological examination disclosed generalized thickening of the skin and a photoaged face. Xanthomatous micropapulous plaques were identified on the forehead, lateral sides of the neck, back, elbows, and knees, which showed a waxy aspect. He had confluent atrophic, acneiform scars on his forehead. On the eyelid borders, it was possible to observe several tiny papules lined up along their margins without eyelash loss, the so-called moniliform blepharitis. In the oral cavity, the tongue showed an irregular depapillated surface, a short frenulum, and also shiny plaques, sometimes whitish and sometimes xanthomatous, on the central portions of the palate (Fig. 1), on the tongue, and the floor of the mouth.

A skin punch biopsy was performed in the cervical region, which was processed for light microscopy (LM), with H&E and PAS staining; a small fragment was dehydrated, for the dermis to be analyzed through scanning electron microscopy (SEM), and another fragment was embedded in resin to obtain ultra-thin sections of the reticular dermis, to be analyzed through transmission electron microscopy (TEM).

## Results

**Figure 2:**
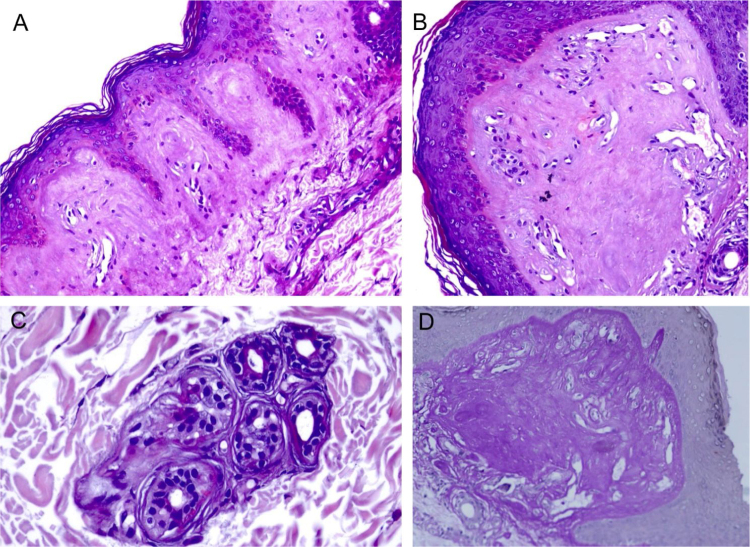
Light microscopy – (A), Eosinophilic deposits in the papillary dermis (Hematoxylin & eosin, ×150); (B), Detail of the papillary dermis with significant homogenization and vascular ectasia (Hematoxylin & eosin, ×400); (C), Discrete deposits around a sweat gland (Hematoxylin & eosin, ×400); (D), PAS staining demonstrating resistant diastase positivity in the papillary dermis (Periodic-acid Schiff ×400).

The LM at a small magnification showed homogenization of the papillary dermis (Fig. 2A), higher magnifications showed intense hyalinization of the papillary dermis, with ectatic vessels (Fig. 2B), also occurring around the sweat glands (Fig. 2C). Dermal deposits were with PAS positive (Fig. 2D) and resistant to diastase.

**Figure 3:**
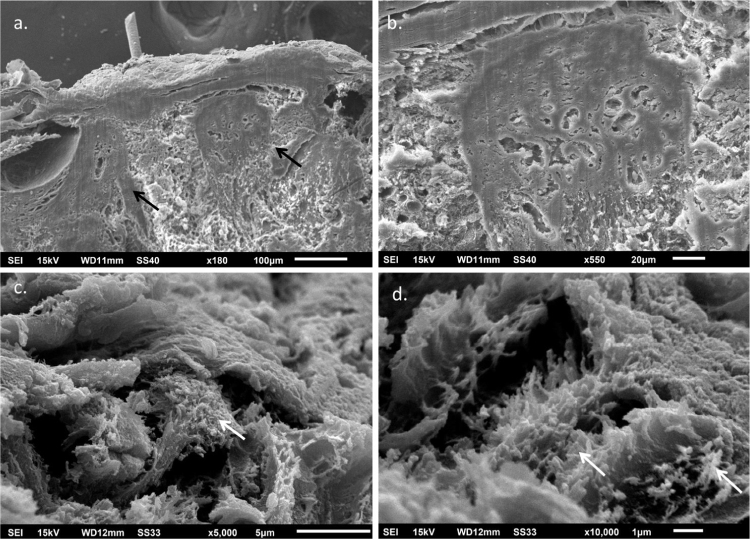
Scanning electron microscopy – (A), Small magnification showing homogenization of the papillary dermis (arrows) (×180); (B), Detail of the compact papillary dermis (×550). (C and D), Fibrillar deposits in the middle dermis (arrows) (×5,000 and 10,000).

The use of SEM at low magnifications demonstrated, similarly to LM, a compact papillary dermis (Fig. 3A), which is very evident at higher magnifications (Fig. 3B). The analysis of the reticular dermis identifies fibrillar deposits, preventing the observation of collagen bundles (Figs. 3C and 3D).

**Figure 4:**
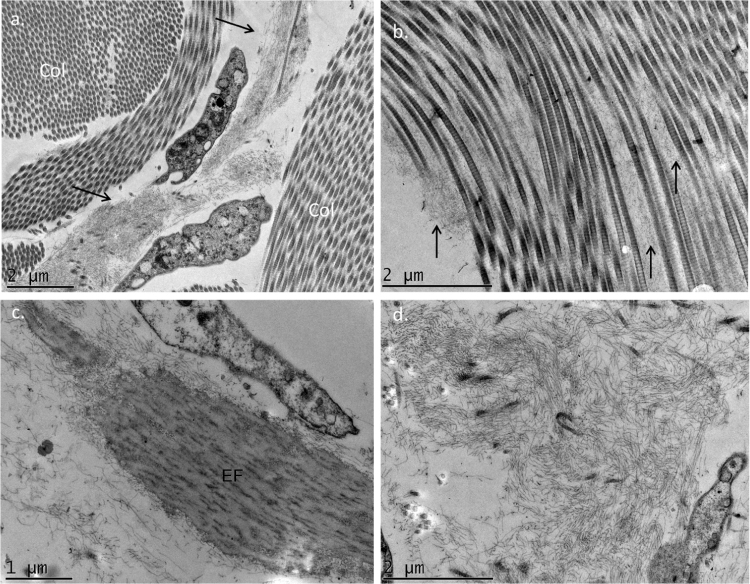
Transmission electron microscopy – (A), Fibrillar material deposits (arrows) between collagen bundles (Col) (×15,000); (B), Fibrillar deposit between collagen fibers (arrows) (×25,000); (C), Fibrillar deposits around an elastic fiber (EF) (×30,000). (D), Area with significant deposition of fibrillar material and displacement of collagen fibers (×25,000).

**Figure 5:**
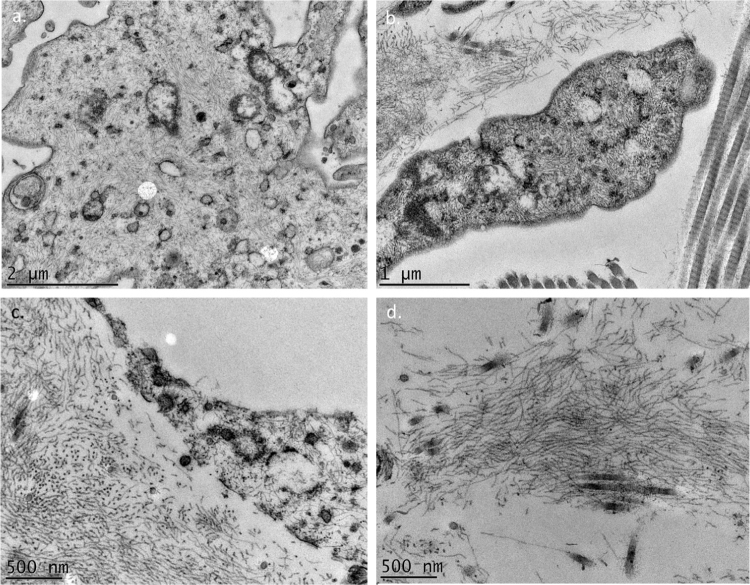
Transmission electron microscopy – (A and B), Presence of fibrillar material inside a cell (×25,000 and ×40,000). (C and D), High magnifications detailing the fibrillar nature of the deposits (×50,000 and ×50,000).

The use of TEM demonstrated, at low magnifications, deposition of fibrillar material between the collagen bundles (Fig. 4A) and the collagen fibers (Fig. 4B), which had a normal morphology. The deposition was also seen adhered to elastic fibers (Fig. 4C) and in larger quantities, in some of the analyzed fields, displacing the collagen fibers (Fig. 4D). Cell structures were also visualized and showed the cytoplasm filled with fibrillar material (Figs. 5A and 5B). At higher magnifications (×50,000) the microfibrillar structure of the deposits is quite evident (Figs. 5C and 5D).

## Discussion

The clinical and light microscopy findings of the case investigated herein overlap with those reported in the literature, with the characteristic changes in the papillary dermis.

No reports were found on the use of SEM in this disease, as this technique is rarely used to investigate the dermis. Similar findings to those of the LM were found in the papillary dermis, which shows a compact aspect in the three-dimensional image. In the middle dermis, the normal components, the collagen bundles, are covered by fibrillar deposits, while with this technique they normally appear as grouped filaments, forming bundles, which look like cords.

Several authors have already reported the deposition of filamentous material when using TEM, and the findings of the present study are similar to those of Hashimoto et al., who described curved or anastomosed filaments (displaying a reticular aspect); these filaments were also seen inside the cells, suggesting a possible source of their origin, as in the case investigated herein. Some authors have described the deposits as granular.^4^, ^5^, ^6^

Biochemical studies suggest the non-collagenic nature of the deposits, in agreement with the present findings, since the collagen is normal, which speaks against a genetic defect in its production, and the deposits morphologically differ from this dermal component.^5^

Epidermal and vascular basement membrane duplication has also been reported; as the present study did not display these structures in the sections, these findings cannot be compared.^7^, ^8^

The exact role of the mutated ECM1 protein in lipoid proteinosis is not well known; it is considered an extracellular matrix glycoprotein.3,^9^ It interacts with several dermal structures, with reports including vascular dermal alterations in the disease, but it is difficult to rule out the possibility that they might be secondary events.^10^

There are four ECM1variants, which makes it even more difficult to correlate mutations with phenotypes and even more so with ultrastructural changes, since there are few reports on the use of electron microscopy, allowing not only a certain phenotypic variability, but also in the ultrastructural morphology of the deposits. With the investigation of more cases, it may be possible to make these correlations in the future.^2^

## Financial support

None declared.

## Authors' contributions

Hiram Larangeira de Almeida Jr: Approval of the final version of the manuscript; study design and planning of the study; drafting and editing of the manuscript; collection, analysis, and interpretation of data; critical review of the literature; critical review of the manuscript.

Raphael Goveia Rodeghiero: Approval of the final version of the manuscript; design and planning of the study; drafting and editing of the manuscript; collection, analysis, and interpretation of data; critical review of the literature; critical review of the manuscript.

Patricia Naomi Ando Suzuki: Approval of the final version of the manuscript; design and planning of the study; drafting and editing of the manuscript; collection, analysis, and interpretation of data; critical review of the literature; critical review of the manuscript.

Marília Marufuji Ogawa: Approval of the final version of the manuscript; design and planning of the study; drafting and editing of the manuscript; collection, analysis, and interpretation of data; critical review of the literature; critical review of the manuscript.

## Conflicts of interest

None declared.

## References

[1] Lima L.R., Mulinari-Brenner F.A., Manfrinato L.C., dal Pizol A.S., Serafini S.Z., Fillus Neto J. (2003). Lipoid proteinosis – a report of two cases. An Bras Dermatol..

[2] Ludew D., Wertheim-Tysarowska K., Budnik K., Grabarczyk A., Kowalewski C., Kapińska-Mrowiecka M. (2018). Lipoid proteinosis: a first report of mutation Val10Gly in the signal peptide of the ECM1 gene. Postepy Dermatol Alergol.

[3] Rey L.K., Kohlhase J., Möllenhoff K., Dekomien G., Epplen J.T., Hoffjan S. (2016). A Novel ECM1 Splice Site Mutation in Lipoid Proteinosis: Case Report plus Review of the Literature. Mol Syndromol.

[4] Hashimoto K., Su W.P., Wang P.W., Eto H. (2000). Late onset hyalinosis cutis et mucosae. J Dermatol.

[5] Fleischmajer R., Krieg T., Dziadek M., Altchek D., Timpl R. (1984). Ultrastructure and composition of connective tissue in hyalinosis cutis et mucosae skin. J Invest Dermatol.

[6] Fabrizi G., Porfiri B., Borgioli M., Serri F. (1980). Urbach-Wiethe Disease. Light and Electron Microscopic Study. J Cutan Pathol.

[7] Mirancea N., Hausser I., Metze D., Stark H.J., Boukamp P., Breitkreutz D. (2007). Junctional basement membrane anomalies of skin and mucosa in lipoid proteinosis (hyalinosis cutis et mucosae). J Dermatol Sci.

[8] Moy L.S., Moy R.L., Matsuoka L.Y., Ohta A., Uitto J. (1987). Lipoid proteinosis: ultrastructural and biochemical studies. J Am Acad Dermatol.

[9] Chan I., Liu L., Hamada T., Sethuraman G., McGrath J.A. (2007). The molecular basis of lipoid proteinosis: mutations in extracellular matrix protein 1. Exp Dermatol.

[10] Kowalewski C., Kozłowska A., Chan I., Górska M., Woźniak K., Jabłońska S. (2005). Three-dimensional imaging reveals major changes in skin microvasculature in lipoid proteinosis and lichen sclerosus. J Dermatol Sci.

